# Postoperative inpatient exercise facilitates recovery after laparoscopic surgery in colorectal cancer patients: a randomized controlled trial

**DOI:** 10.1186/s12876-023-02755-x

**Published:** 2023-04-17

**Authors:** Jihee Min, Ki-yong An, Hyuna Park, Wonhee Cho, Hye Jeong Jung, Sang Hui Chu, Minsoo Cho, Seung Yoon Yang, Justin Y. Jeon, Nam Kyu Kim

**Affiliations:** 1 National Cancer Survivorship Center, National Cancer Control Institute, National Cancer Center, Goyang-si, Republic of Korea; 2 Department of Sport Industry Studies, Exercise Medicine and Rehabilitation Laboratory, Yonsei University, Seoul, Republic of Korea; 3 Faculty of Kinesiology, Sport, and Recreation, University of Alberta, Edmonton, Alberta Canada; 4 Department of Nursing, Mo-Im Kim Nursing Research Institute, Yonsei University, Seoul, Republic of Korea; 5 Department of Surgery, Yonsei University College of Medicine, Seoul, Republic of Korea; 6 Exercise Medicine Center for Diabetes and Cancer Patients, Yonsei University, Seoul, Republic of Korea; 7 Cancer Prevention Center, Yonsei Cancer Center, Shinchon Severance Hospital, Seoul, Republic of Korea; 8 Department of Sports Industry Studies, Yonsei University, Seoul, South Korea

**Keywords:** Exercise, Colectomy, Colorectal cancer, Post-operative, Length of stay, Patient-perceived readiness for hospital discharge

## Abstract

**Background:**

Early mobilization is an integral part of an enhanced recovery program after colorectal cancer surgery. The safety and efficacy of postoperative inpatient exercise are not well known. The primary objective was to determine the efficacy of a postoperative exercise program on postsurgical recovery of stage I–III colorectal cancer patients.

**Methods:**

We randomly allocated participants to postoperative exercise or usual care (1:1 ratio). The postoperative exercise intervention consisted of 15 min of supervised exercise two times per day for the duration of their hospital stay. The primary outcome was the length of stay (LOS) at the tertiary care center. Secondary outcomes included patient-perceived readiness for hospital discharge, anthropometrics (e.g., muscle mass), and physical function (e.g., balance, strength).

**Results:**

A total of 52 (83%) participants (mean [SD] age, 56.6 [8.9] years; 23 [44%] male) completed the trial. The median LOS was 6.0 days (interquartile range; IQR 5–7 days) in the exercise group and 6.5 days (IQR 6–7 days) in the usual-care group (*P* = 0.021). The exercise group met the targeted LOS 64% of the time, while 36% of the usual care group met the targeted LOS (colon cancer, 5 days; rectal cancer, 7 days). Participants in the exercise group felt greater readiness for discharge from the hospital than those in the usual care group (Adjusted group difference = 14.4; 95% CI, 6.2 to 22.6; *P* < 0.01). We observed a small but statistically significant increase in muscle mass in the exercise group compared to usual care (Adjusted group difference = 0.63 kg; 95% CI, 0.16 to 1.1; *P* = 0.03).

**Conclusion:**

Postsurgical inpatient exercise may promote faster recovery and discharge after curative-intent colorectal cancer surgery.

**Trial registration:**

The study was registered at WHO International Clinical Trials Registry Platform (ICTRP; URL http://apps.who.int/trialsearch); Trial number: KCT0003920.

**Supplementary Information:**

The online version contains supplementary material available at 10.1186/s12876-023-02755-x.

## Background

Globally, colorectal cancer is the third most commonly diagnosed cancer and the second leading cause of cancer death [1]. Surgery is the primary treatment for colorectal cancer. Colorectal cancer surgery is associated with a prolonged hospital stay, postoperative ileus, declined physical function, and possible surgical site infection [2, 3]. Laparoscopic surgery for colorectal cancer is practiced widely with patient benefits, including reduced hospital stay, earlier return of bowel function, better pulmonary function, and reduced morbidity compared to open surgery [4].

After laparoscopic surgery, primary care includes standardized perioperative practices known as an enhanced recovery program [5]. Research has suggested enhanced recovery programs (ERP)s may improve surgical outcomes and prevent complications, decrease hospital length of stay (LOS), reduce healthcare costs, and improve patient satisfaction [5–9]. While early mobilization may reduce LOS [10, 11], evidence of beneficial impacts of early mobilization on surgical outcomes is insufficient for guiding best clinical practices [5, 12–14]. Early mobilization protocols vary across studies [14–18]. For example, one protocol included being out of bed for > 8 h per day on postoperative day 1 [15], while another protocol included sitting on a chair for > 1 h per day with ambulation for > 400 m on postoperative day 1 [16]. Studies have also reported that walking as a postoperative mobilization strategy does not result in a significant reduction in LOS [17, 18].

Our previous work demonstrated patients who received tailored exercise programs, including stretching, strengthening, balance, and walking exercises, had a significantly shorter LOS (7.82 ± 1.07 vs. 9.86 ± 2.66 days) than those who did not [19]. The study had a small sample size and was performed without applying other components of ERP. Knowing that most clinics use some form of ERP, it was imperative to repeat the study with a large sample of participants who received an ERP. Since an ERP encourages a predetermined LOS for different surgeries, it was important also to assess the patient’s perceived readiness for hospital discharge. The primary objective of this randomized controlled trial was to examine the effect of postoperative inpatient exercise on LOS in patients who had undergone laparoscopic colorectal cancer surgery. The secondary objective was to examine the effect of postoperative exercise on patients’ perceived readiness for hospital discharge.

## Methods

### Study Design and participants

This single-center randomized controlled trial was conducted at the Colorectal Cancer Clinic, a tertiary referral center in Seoul, Korea. We recruited participants between February 5, 2014 and September 23, 2016. Inclusion criteria were (1) stage I-III colon or rectal cancer; (2) 19–70 years of age; (3) American Society of Anesthesiologist grade ≤ 3 at surgery; and (4) the ability to read and understand Korean. Primary exclusion criteria included (1) evidence of recurrent or metastatic disease; (2) postsurgical intensive care unit stay; (3) presence of a stoma after colectomy; and (4) open surgery. All patients who met our inclusion criteria were approached, and those who agreed to participate were recruited into our study. The Institutional Ethics Review Board of Severance Hospital approved the trial (IRB No. 4-2013-0868, 02/05/2014) and the trial was registered at http://apps.who.int/trialsearch (trial number: KCT0003920, 05/15/2019) and conforms to CONSORT guidelines for randomized controlled trials. The eligible participants provided informed consent before the initiation of any study-related procedures.

### Outcomes and data Collection

The primary outcome of this study was LOS, defined as the *number of days between the day of surgery and the day of discharge*. Patients were discharged when the following conditions were met: (1) vital sign stability (blood pressure, body temperature, pulse rate, and respiration rate); (2) soft diet tolerance; (3) clearing of the wound; (4) pain control; (5) no difficulty of voiding; and (6) passage of first stool. Target LOS duration for the current ERP was 5 days for colon cancer and 7 days for rectal cancers.

The secondary outcomes included patient-perceived readiness for hospital discharge (Pt-RHDS). The Pt-RHDS [20, 21] was translated into Korean and back-translated by two independent bilingual scholars to examine the construct validity among Korean colorectal cancer patients. After excluding a question with a similar meaning as another question, the Korean version of the Pt-RHDS questionnaire comprised 22 items that measured four domains. *Personal status* measured how the patient feels on the day of discharge. *Knowledge* measures the patients’ knowledge about self-care at home after discharge. *Coping ability* measured how the patient will be able to cope at home after discharge. Finally, *expected support* measured how much help the patient will have if/when needed at home after discharge). Each question was scored from 0 (not at all) to 10 (absolutely), and higher scores were interpreted as greater readiness for hospital discharge. To facilitate interpretation, the total score and each RHDS domain were converted to a standardized 100-point scale so that the maximum possible score at each level was 100. The internal consistency of the questionnaire was evaluated using Cronbach’s coefficient, and it was 0.891 for the total scale in this study (previously studied was reported as 0.90 [21]). The validity of the questionnaire was internally validated (face and content validity) and tested in colorectal cancer patients.

We measured other secondary outcomes including anthropometric measures such as height, weight, waist circumference, and thigh circumference. We measured waist circumference using medical body measuring tape at the midpoint between the lower border of the 12th rib cage and the iliac crest. We measured thigh circumference at the midpoint of the thigh. The body composition including body mass index, muscle mass, and fat mass was assessed using bioelectrical impedance analysis (BIA) (Inbody 230, Biospace, Seoul, South Korea). Tests were completed on the day before the surgery and the day of hospital discharge (i.e., twice). Physical function measures included handgrip strength using a grip dynamometer (GRIP-D 5401, TKK, Japan), lower body strength (chair-stand test) and balance ability (time participant could stand on one leg).

### Sample size calculation

To detect an effect size (Cohen’s d = 0.8), we initially calculated a required sample size of 52. However, anticipating an expected dropout rate of 20%, we enrolled a final sample size of 64 (32 per group) in our study. Our analysis identified an effect size of d = 0.8 with 0.80 power and a two-tailed overall type I error rate of 0.05. We based our power calculation on a previous study with a similar exercise protocol and primary outcome measure in colorectal cancer patients, which reported an effect size of 0.77 (LOS = 7.82 ± 1.07 days in the exercise group, 9.86 ± 2.66 days in the usual care group) [19].

### Randomization

A total of 124 patients who met inclusion criteria were assessed for eligibility. Among them 60 patients were excluded due to inability to understand Korean, 2 medical issues, and 57 declined participation of the study. Out of 64 patients, we excluded one participant before randomization due to metastasis found during surgery. A total of 63 patients were randomly assigned to the exercise or usual care groups (1:1 ratio). Randomization was performed using a permuted block design, with stratification by age, sex, body mass index, and cancer type (colon vs. rectal). Allocation concealment was implemented by sequentially numbered, sealed and opaque envelopes. We used a minimization method to balance the prognostic factors between the groups [22, 23]. Study staffs were not blinded to treatment assignment. Among the patients randomized, 11 (5 in the exercise group and 6 in the usual care group) were randomized but excluded from the trial due to ileostomy and did not receive any intervention.

### Study groups

#### Exercise intervention

We implemented the institutional ERP protocol for all trial participants. The details of the ERP protocol are attached as supplementary material (Supplementary Table 1). Participants randomized to the exercise group engaged in a 15-minute supervised exercise intervention twice a day. A qualified exercise specialist under the guidance of a professor in the field of exercise oncology provided the exercise intervention. The exercise program consisted of three phases according to postoperative day and patients’ conditions.

The phase 1 exercise program started on postoperative day 1 (patient condition: patients experienced limited mobility due to pain; patients required help because of pain at the surgical site when sitting or lying down). During this phase, the exercises consisted of stretching and low-intensity resistance exercises (i.e., stretching the neck, shoulder, wrist, and ankle, pelvic stretching, and posterior pelvic tilt). We implemented phase 2 on postoperative days 2 and 3 (patient condition: patients able to perform daily activities without help or to be able to walk for > 20 min at a time, but still experiencing discomfort). In this phase, in addition to phase 1 exercises, participants performed stretching, resistance exercises (i.e., leg raise, leg circle, bridge, and squeezing a ball with the thighs) and core resistance exercises (i.e., including arm circles, triceps extensions, and posterior pelvic tilt in the supine position). The phase 3 exercise program was performed between postoperative day 4 to hospital discharge (patient condition: can perform daily activities and self-care activities without discomfort. Phase 3 program was a continuation of Phase 2, with balancing exercises including one-leg standing, one-leg calf raises, hip adduction, hip abduction, hip extension, and hip flexion. Each movement was performed in sets of 10 repetitions for isotonic exercises or 10 s for isometric exercises.

The participants in the usual care group received the same preoperative and postoperative ERP as the exercise group (Supplementary Table 1). The usual care group did not receive the exercise program.

### Statistical analysis

We used descriptive statistics describe and summarize the study sample. Primary and secondary analyses used intention-to-treat (ITT) analyses using multiple-imputation with expectation-maximization algorithm methods [24]. Multiple-imputation methods were used for two participants in the exercise group and one in the control group for RHDS measures. Per-protocol analyses were performed for sensitivity analyses (Supplementary Table 2). Data normality was examined using the Kolmogorov-Smirnov test before initiating any statistical analyses. A chi-square test or independent t-test was used to test for differences between groups at baseline. All study outcomes were compared using analysis of covariance (after adjusting for sex, cancer type and stage), or the Wilcoxon signed-rank test or Mann-Whitney U-test, depending on the distribution of the data. When analysis of covariance was used, data was presented as mean ± standard deviation. When Mann- Whitney U-test was used, data was presented as median with interquartile range (IQR). Statistical significance was set at *P* < 0.05, and data were analyzed using SPSS version 20 (IBM Corp., Armonk, NY, USA).

## Results

**Fig. 1:**
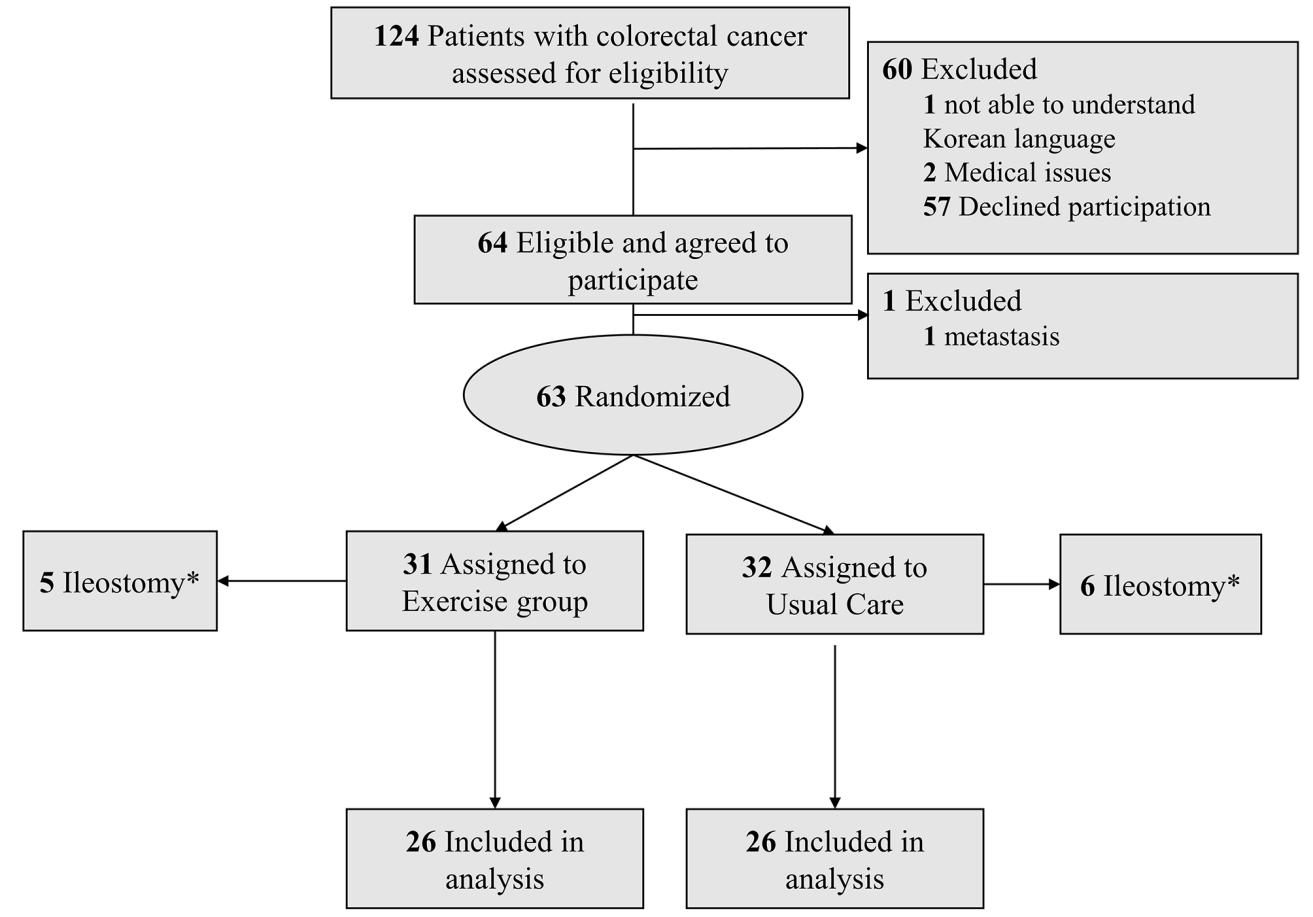
Study Flow Diagram * Patients assigned to group but did not receive treatment and excluded from the analyses

A total of 52 participants completed the trial (see Fig. 1 for study flow). Participants in the exercise intervention completed 83.6% of exercise sessions. Table 1 shows the participants’ baseline demographic and medical profiles across each group. The mean age of the participants was 56.6 (8.9) years (exercise, 56.8 ± 7.7 years vs. usual care, 56.4 ± 9.6 years, *P* = 0.87). Cancer diagnoses were evenly split between colon (53.8%) and rectal (46.2%). The average surgery time was 4.8 ± 1.8 h. There was no significant difference in any baseline characteristics across the groups.

**Table 1:** Participants’ Demographic and Clinical Characteristics

Demographic and Clinical Variables	Total (n = 52)	Exercise (n = 26)	Usual Care (n = 26)	*P* value
Age, mean (SD)	56.6 (8.9)	56.8 (7.7)	56.4 (9.6)	0.87
Weight, mean (SD), kg	62.3 (9.4)	60.6 (10.3)	64 (8.3)	0.20
Body mass index, mean (SD), kg/m^2^	23.1 (2.7)	22.9 (2.8)	23.3 (2.6)	0.60
Males (%)	23/52 (44.2)	10/26 (38.5)	13/26 (50)	0.58
Cancer type (%)				
Colon	28/52 (53.8)	14/26 (53.8)	14/26 (53.8)	1.00
Rectal	24/52 (46.2)	12/26 (46.2)	12/26 (46.2)	
Cancer stage (%)				
I	19/52 (36.5)	8/26 (30.8)	11/26 (42.3)	0.49
II	15/52 (28.8)	7/26 (26.9)	8/26 (30.8)	
III	18/52 (34.6)	11/26(42.3)	7/26 (26.9)	
Operation type (%)				
Low anterior resection	9/52 (17.3)	5/26 (19.2)	4/26 (15.4)	0.41
Anterior resection	31/52 (59.6)	15/26 (57.7)	16/26 (61.5)	
Right hemicolectomy	11/52 (21.2)	6/26 (23.1)	5/26 (19.2)	
Left hemicolectomy	1/52 (1.9)	0	1/26 (3.8)	
Methods of surgery (%)				
Laparoscopic surgery	44/42 (84.6)	23/26 (88.5)	21/26 (80.8)	0.71
Robot surgery	8/52 (15.4)	3/26 (11.5)	5/26 (19.2)	
Duration of surgery, mean (SD), hours	4.8 (1.8)	4.8 (2.1)	4.8 (1.6)	1.00
Surgical complications (%)	3/52 (5.8)	2/26 (7.7)	1/26 (3.8)	0.55

Abbreviations: SD, standard deviation

### Primary outcome

**Fig. 2:**
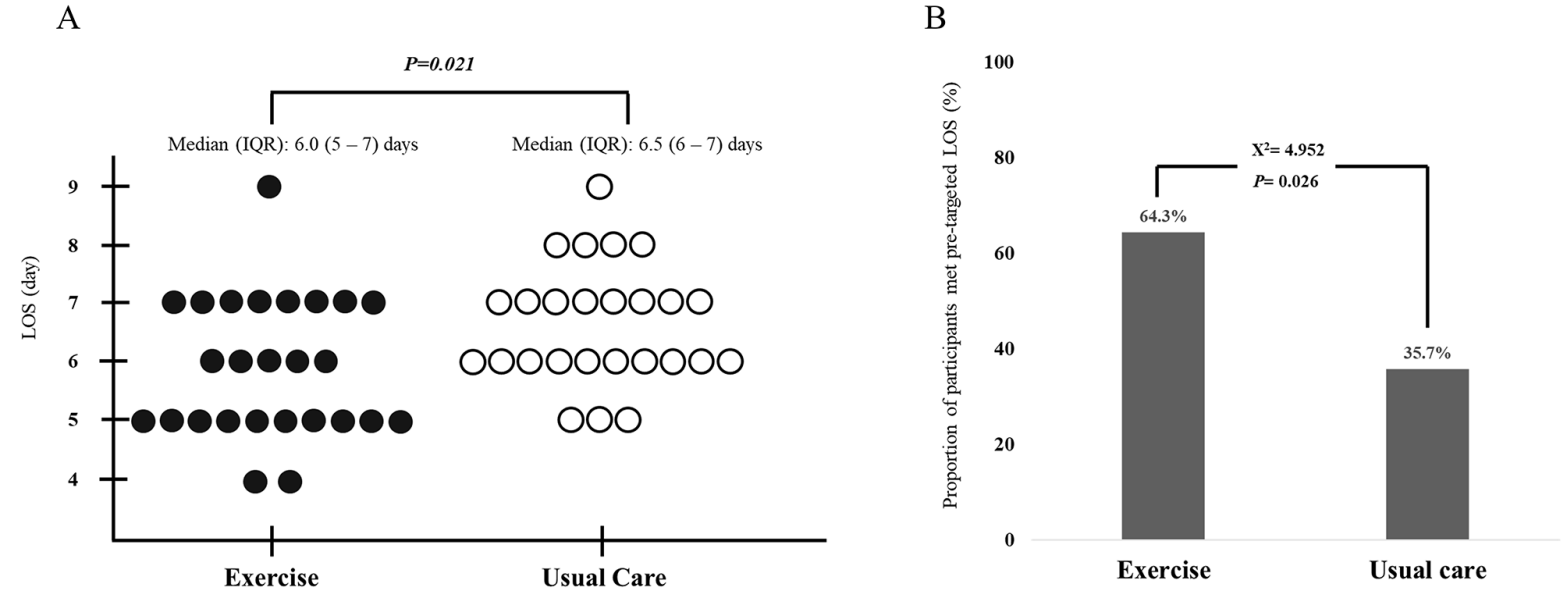
Effects of Postoperative Exercise on Length of Stay (**A**) Data were presented as median and interquartile range (IQR). Mann Whitney U-test was employed since data was not normally distributed. Abbreviation: length of stay (LOS). The mean ± SD was 5.81 ± 1.01 days in the exercise group and 6.63 ± 1.02 days in the usual care group (**B**) The target LOS for the ERAS protocol used in this study was colon cancer (≤ 5 days) and rectal cancer (≤ 7 days)

Median LOS was significantly longer for participants in the usual care group. The median LOS was 6.0 days (IQR 5–7 days) in the exercise group and 6.5 days (IQR 6–7 days) in the usual-care group (*P* = 0.021; Fig. 2A). A higher proportion of participants in the exercise group met the target LOS compared to participants in the usual care group (exercise group: 64% vs. usual care 36%; *P* = 0.026; Fig. 2B). Reduced LOS was observed across both cancer types (i.e., colon and rectal) (Supplementary Table 3).

### Secondary outcomes

Result for all secondary outcomes are shown in Tables 2 and 3. Participants in the exercise group felt greater readiness for discharge from the hospital (i.e., Pt-RHDS scores) compared with those in the usual care group (Adjusted group difference = 14.4; 95% CI, 6.2 to 22.6; *P* < 0.01). Participants in the exercise group also reported significantly higher scores on personal status compared to those in the usual care group (Adjusted group difference = 6.9; 95% CI, 0.6 to 13.2; *P* < 0.05). Compared to the usual care group, we observed small but statistically significant increase in the exercise group on muscle mass (Adjusted group difference = 0.63 kg; 95% CI, 0.16–1.09; *P* = 0.03), balance (Adjusted group mean = 2.8 s; 95% CI, 0.2 to 5.3; *P* = 0.04), and chair-stand test results (Adjusted group mean = 2.2 beats per minute; 95% CI, 1.4 to 3; *P* < 0.01).

**Table 2:** Effects of Postoperative Exercise on Patient-Perceived Readiness for Hospital Discharge

Outcome	Exercise (n = 26)	Usual Care (n = 26)	Mean difference between group	*P* value
Readiness for discharge^a^	82.5 (16.9)	68.5 (22.3)	14.4 (6.1 to 22.6)	< 0.01
Personal status^b^	59.1 (14)	53.3 (14.1)	6.9 (0.6 to 13.2)	0.03
Knowledge^b^	52.9 (29)	57.8 (25.1)	-2.3 (-13.6 to 8.7)	0.66
Coping ability^a^	69.8 (18.4)	68.7 (18.6)	1.0 (-6.6 to 8.6)	0.88
Expected support^a^	71.4 (16.6)	68.5 (20.7)	2.9 (-5.8 to 11.5)	0.60

Values are presented as mean (SD) or mean and 95% confidence interval (CI).

^a^Mann-Whitney U-test was used as the data were not normally distributed

^b^Adjusted for cancer stage and sex

**Table 3:** Effects of Postoperative Exercise on Body Composition and Fitness Outcomes

Outcome	Exercise (n = 26)		Usual Care (n = 26)		Mean difference between group	
	Baseline	Discharge	Baseline	Discharge	Mean (95% CI)	*P* value
Body composition						
Weight, mean (SD), kg^b^	60.6 (10.3)	59.8 (9.6)^**^	64 (8.3)	63.2 (8.4)^*^	0.18 (-0.47 to 0.84)	0.58
Body mass index, mean (SD), kg/m^2b^	22.9 (2.8)	22.6 (2.6)^**^	23.3 (2.6)	23.1 (2.7)	0.04 (-0.21 to 0.28)	0.76
Muscle mass, mean (SD), kg^a^	24.2 (5.3)	24.5 (5.2)^*^	26.3 (4.8)	25.7 (4.6)	0.63 (0.16 to 1.09)	< 0.01
Percent body fat (%)^b^	27 (5.6)	25 (5.9)^***^	25.4 (7.7)	25.3 (7.5)	-0.91 (-1.86 to 0.04)	0.06
Waist circumference, mean (SD), cm^b^	81.2 (9.9	81.5 (9.1)	81.8 (8.2)	83.1 (7.7)	0.17 (-1.09 to 1.43)	0.78
Thigh circumference, mean (SD), cm^b^	48.9 (3.6)	48.8 (3.7)	48.2 (4.4)	47.3 (4.2)^*^	0.79 (-0.20 to 1.77)	0.11
Physical function test						
Shoulder flexibility, mean (SD), cm^a^	-6.2 (12.3)	-5.5 (11.2)	-12.5 (11.5)	-12.9 (11.2)	1.36 (-0.28 to 2.99)	0.10
Balance, mean (SD), seconds^a^	24.2 (9.1)	25.7 (8)	23.9 (9.3)	22.6 (8.4)	2.77 (0.21 to 5.34)	0.04
Hand grip, mean (SD), kg^a^	27.1 (9)	26.7 (8.9)	30.8 (11.8)	29.2 (11.1)^*^	1.04 (-0.53 to 2.61)	0.19
Chair stand, mean (SD), repetitions^a^	12.9 (2.5)	14 (2.6)^*^	12.7 (2.8)	11.1 (2.6)^**^	2.22 (1.41 to 3.04)	< 0.01

Values are presented as mean (SD) or mean and 95% confidence interval (CI).

Abbreviations: CM, centimeters; SD, standard deviation

*P < 0.05, **P < 0.01, ***P < 0.001

^a^Wilcoxon Signed Rank-test was used as the data were not normally distributed

^b^Adjusted for cancer stage and sex

### Adverse events

No exercise related injury is reported during hospitalization. We followed up with the participants for 30 days after discharge and observed no hospital readmissions in either group. During this period, we noted one case of atelectasis and polyuria in the exercise group and urinary retention in the control group, which were successfully treated in our outpatient clinic. The incidence of complications was not significantly different between the groups (*P* = 0.55).

## Discussion

In this randomized controlled trial, we examined the effect of postoperative inpatient exercise on LOS, perceived readiness for discharge, and other physical and anthropometric outcomes (e.g., weight, balance). We observed a significant reduction in hospital LOS and higher perceived readiness for hospital discharge among the participants in the exercise group suggesting the beneficial effect of exercise for recovery after colorectal cancer surgery. We also found participants who exercised improved their physical function and were better able to maintain their muscle mass. We observed that implementing a postoperative exercise among colorectal cancer patients improved the recovery after colorectal cancer surgery and prepared patients for hospital discharge.

We observed a median LOS of 6.0 days in the exercise and 6.5 days in the usual care groups. Participants in the exercise group were discharged over half a day earlier compared to usual care participants. Our analysis also showed 64% in the exercise group met the target LOS goal while only 36% in the usual care met this goal. Those in the exercise group were not only discharged from the hospital early, but they indicated they felt they were more ready to be discharged from the hospital at the time of hospital discharge compared to usual care participants. Since the ERP protocol is a multimodal approach to enhance recovery after surgery and often aims to shorten the LOS [25], perceptions about readiness for discharge are an important variable. When participants in our study were asked “How ready are you to be discharged from the hospital?”, the exercise group scored 15% higher compared with participants in the usual care group. Participants in the usual care group stayed in the hospital longer and still felt less ready to be discharged. Shorter LOS after surgery is associated with increased readmission after hospital discharge [26–28]. In our study, participants in the exercise group scored higher in personal status (how a patient feels on the day of discharge). With significantly reduced LOS and higher Pt-RHDS without increased hospital readmissions (as seen in previous research) among participants in the exercise group, our study suggests supervised inpatient exercise after surgery helped participants recover from the surgery. Future research should determine the cost savings associated with these exercise programs.

Contextual factors may help explain the observed reduction in LOS. When home care service after discharge (e.g., provided by nurses, physiotherapists, social workers) is part of routine medical care, early discharge from the hospital after surgery can be safe and effective [29, 30]. When providing home care service is not feasible, clinical staff (e.g., surgeons) have to ensure patients are ready to be discharged without any major side effects or elevated risk of readmission. As a result, LOS is often longer in settings where home care service is not part of routine clinical practice [31–34]. Our trial took place in a tertiary care center in Seoul, South Korea, where home care service is *not a part of routine medical practice* and LOS is relatively longer. Whether postoperative exercise was provided during the hospital stay or after hospital discharge, the shorter LOS among patients in the exercise group demonstrated the beneficial impact of exercise on patient recovery. We believe the findings from the current study are clinically meaningful and suggest that exercise targeted to patients’ conditions should be a part of the ERP, and maintained after discharge from the hospital.

This trial adds to a body of literature that has yielded conflicting results. Several studies reported no effect of early mobilization on LOS focused on walking as a mode of early mobilization [17, 18]. Recently, Onerup A et al. [35] reported lack of short-term homebased pre- and postoperative exercise on recovery after colorectal cancer surgery. Authors applied pre- and postoperative exercise on the intervention group while same early mobilization mostly composed of walking and breathing exercise were applied both intervention and control group. In a small sample of 31 colon cancer patients, Ahn et al. [19] tested a supervised exercise program similar to ours in the postsurgical context. Their program included twice daily, 15-minute sessions of stretching and low intensity resistance exercises, and progressed to strengthening and balancing exercises. Ahn et al. [19] reported the usual care group had a higher average walking distance per day than the exercise group, suggesting that walking may not influence LOS as much as resistance-based exercises. The trial by Ahn et al. [19] did not implement an ERP, and their pre-target LOS was longer than the targets in our trial.

We need to consider limitations to our trial when drawing conclusions. First, the duration of the exercise intervention during the hospital stay was relatively short. Secondly, we could not assess whether the observed changes in the outcomes were maintained after hospital discharge. As this was a single-center study, caution should be taken when generalizing our findings to the broader population of colorectal cancer survivors. Since the total body water content may influence the result of BIA measurements, BIA for measuring muscle mass during hospitalization (mainly when intravenous fluid regulation is applied) may not be the ideal choice. Given the same condition (i.e., hydration) was used both pre and post-intervention in both the exercise and control groups may increase the reliability of our measurements. Strengths of our trial include the objective assessments of several of our outcomes (e.g., muscle mass, balance) and the randomized controlled trial design. While multi-center trials are desirable, our single-site trial allowed for consistency in the surgery protocol, technique, and ERP and exercise intervention delivery.

In conclusion, our trial demonstrated postsurgical inpatient exercise may promote faster recovery and discharge after curative colorectal cancer surgery. We observed that participants in the exercise group had significantly shorter LOS and stronger perceptions that they were ready for discharge from the hospital compared to the usual care group. Future research should continue to (1) examine the impact of exercise on hospital length of stay in the colorectal cancer context; (2) determine the role of other modes of exercise (e.g., walking, stationary cycling, yoga, resistance training), and even compare their associations with LOS at this point in the cancer trajectory; (3) determine the cost savings associated with exercising after surgery. Future multi-center trials with larger sample sizes and longer follow-up will facilitate a better understanding of this area.

## Electronic supplementary material

Below is the link to the electronic supplementary material.

Supplementary Material 1

Supplementary Material 2

Supplementary Material 3

Supplementary Material 4

## Data Availability

The datasets used and/or analyzed during the current study are available from the corresponding author on reasonable request.

## List of abbreviations

LOS: Length of stay
Pt-RHDS: Patient-perceived readiness for hospital discharge
ERPs: Enhanced recovery programs
BIA: Bioelectrical impedance analysis.
