# Supplementary material for: Postoperative inpatient exercise facilitates recovery after laparoscopic surgery in colorectal cancer patients: a randomized controlled trial
Source: BMC Gastroenterol. 2023 Apr 17;23:127. doi: 10.1186/s12876-023-02755-x (PMC10111844; doi:10.1186/s12876-023-02755-x)
Supplement: Supplementary file 1 — Supplementary Material 1 [file 12876_2023_2755_MOESM1_ESM.doc]

Table S1. Enhanced Recovery Program (ERP)

| **Period** | **Component** | **Content** |
| --- | --- | --- |
| **Preoperative** | |  |
|  | Vital sign (V/S) | Blood pressure, body temperature (BT), pulse rate, respiration rate |
|  | Preoperative oral carbohydrate intake | White porridge, midnight: nil per os (NPO) |
|  | Bowel preparation | Cleansing & shaving, colyte 4L (4hours), intravenous (IV) fluid |
|  | Check medication | blood pressure (BP) medicine, cardiovascular medicine (cardio), diabetes medicine (DM), and anticoagulant |
|  | Antibiotics prophylaxis | be initiated within one hour before surgical incision |
|  | Patient education | The operation, watching DVD [respiration exercise, coughing, early exercise (ambulating), pain management (patient-controlled analgesia; PCA)] |
| **Intraoperative** | |  |
|  | Vital sign (V/S) | Blood pressure (BP), body temperature (BT), pulse rate, respiration rate |
|  | Diet | NPO |
|  | Epidural or spinal anesthesia |  |
|  | Body temperature preservation | Use of air warmer and transesophageal monitoring device |
|  | Restrictive fluid strategy | Crystalloid 2-4mL/hr |
|  | Postoperative nausea and vomiting (PONY) prophylaxis | Administered before and of surgery |
| **Postoperative** | |  |
|  | Monitored patients’ status | BT, total input/output, urine output, hemo-vac (HV) color, visual analogue scale (VAS), check PCA |
|  | O_2_ stop | Util the postoperative day (POD) 1 (maximum 2L; a.m. 6) |
|  | SCD stop | Util the POD 1 (a.m. 10) |
|  | Balanced fluids | Daily total fluid level 500ml or less |
|  | Remove urinary drainage | Withdrawal of foley catheter before POD2  If self-voiding was difficult, conducted nelation catheter |
|  | Postoperative epidural analgesia | Using at least two days of PCA through epidural route |
|  | Stimulation of gut motility | Laxatives, digestive medicine used at POD 2 |
|  | Termination of IV fluid infusion | Until the POD3 |
|  | Effective pain control | Well-controlled pain with the nonsteroidal anti-inflammatory drug |
|  | Remove drainage | Until the POD4 (colon), or POD 5 (rectal) |
|  | Diet | POD1: peripheral parenteral nutrition (PPN) starts and S. O. W  POD2: full liquid diet (FLD)  POD3: soft diet |
|  | Mobilization | On the day of surgery: semi-fowler’s position  POD1~dischage at the hospital: recommend walking |
|  | Education | respiration exercise, coughing, early exercise (ambulating), pain management |
