# Supplementary material for: Postoperative inpatient exercise facilitates recovery after laparoscopic surgery in colorectal cancer patients: a randomized controlled trial
Source: BMC Gastroenterol. 2023 Apr 17;23:127. doi: 10.1186/s12876-023-02755-x (PMC10111844; doi:10.1186/s12876-023-02755-x)
Supplement: Supplementary file 2 — Supplementary Material 2 [file 12876_2023_2755_MOESM2_ESM.doc]

Table S2. Effects of Postoperative Exercise on Length of Stay (Per Protocol analysis)

|  | **Exercise (n=24)** | **Usual Care (n=25)** | ***P* value** |
| --- | --- | --- | --- |
| Length of stay (days) | 5.5 (5-7) | 7 (6-7) | **0.023** |
| The variables are presented as median and interquartile range (IQR).  Mann Whitney U-test was employed since the data were not normally distributed.  The mean±SD of LOS was 5.88±1.23 days in the exercise group and 6.64±1.04 days in the usual care group. | | | |
