# Supplementary material for: Postoperative inpatient exercise facilitates recovery after laparoscopic surgery in colorectal cancer patients: a randomized controlled trial
Source: BMC Gastroenterol. 2023 Apr 17;23:127. doi: 10.1186/s12876-023-02755-x (PMC10111844; doi:10.1186/s12876-023-02755-x)
Supplement: Supplementary file 3 — Supplementary Material 3 [file 12876_2023_2755_MOESM3_ESM.doc]

Table S4. Effects of Postoperative Exercise on Time to Flatus

| **Variable** | **Exercise (n=26)** | **Usual Care (n=26)** | ***P* value** |
| --- | --- | --- | --- |
| Time to flatus (hours) | 55.55 (11.83) | 54.53 (23.36) | 0.84 |
| The values are presented as mean (SD). | | | |
