# Supplementary material for: Postoperative inpatient exercise facilitates recovery after laparoscopic surgery in colorectal cancer patients: a randomized controlled trial
Source: BMC Gastroenterol. 2023 Apr 17;23:127. doi: 10.1186/s12876-023-02755-x (PMC10111844; doi:10.1186/s12876-023-02755-x)
Supplement: Supplementary file 4 — Supplementary Material 4 [file 12876_2023_2755_MOESM4_ESM.doc]

Table S3. Effects of Postoperative Exercise on Length of Stay According to Cancer Type (ITT)

| **Length of stay (days)** | **Exercise** | **Usual Care** | ***P* value** |
| --- | --- | --- | --- |
| Colon | (n=14) | (n=14) |  |
|  | 6 (5-7) | 6.5 (6-7) | 0.19 |
| Rectal | (n=12) | (n=12) |  |
|  | 5.5 (5-7) | 6.5 (6-8) | 0.06 |
| The variables are presented as median and interquartile range (IQR).  Mann Whitney U-test was employed since the data were not normally distributed.  The mean±SD of LOS was colon: 5.93±1.11 days in the exercise group and, 6.43±0.85 days in the usual care group, rectal: 5.83±1.11 days in the exercise group and, 6.83±1.19 days in the usual care group. | | | |
